# Prediction of long-term hospitalisation and all-cause mortality in patients with chronic heart failure on Dutch claims data: a machine learning approach

**DOI:** 10.1186/s12911-021-01657-w

**Published:** 2021-11-01

**Authors:** Onno P. van der Galiën, René C. Hoekstra, Muhammed T. Gürgöze, Olivier C. Manintveld, Mark R. van den Bunt, Cor J. Veenman, Eric Boersma

**Affiliations:** 1 Zilveren Kruis Achmea, Zeist, The Netherlands; 2 Department of Cardiology, Thorax Centre, Erasmus MC, University Medical Centre Rotterdam, Rotterdam, The Netherlands; 3 TNO, Leiden, The Netherlands; 4 Leiden University, Leiden, The Netherlands

**Keywords:** Heart failure, Health insurance claims, Prognosis, Outcomes, Machine learning

## Abstract

**Background:**

Accurately predicting which patients with chronic heart failure (CHF) are particularly vulnerable for adverse outcomes is of crucial importance to support clinical decision making. The goal of the current study was to examine the predictive value on long term heart failure (HF) hospitalisation and all-cause mortality in CHF patients, by exploring and exploiting machine learning (ML) and traditional statistical techniques on a Dutch health insurance claims database.

**Methods:**

Our study population consisted of 25,776 patients with a CHF diagnosis code between 2012 and 2014 and one year and three years follow-up HF hospitalisation (1446 and 3220 patients respectively) and all-cause mortality (2434 and 7882 patients respectively) were measured from 2015 to 2018. The area under the receiver operating characteristic (ROC) curve (AUC) was calculated after modelling the data using Logistic Regression, Random Forest, Elastic Net regression and Neural Networks.

**Results:**

AUC rates ranged from 0.710 to 0.732 for 1-year HF hospitalisation, 0.705–0.733 for 3-years HF hospitalisation, 0.765–0.787 for 1-year mortality and 0.764–0.791 for 3-years mortality. Elastic Net performed best for all endpoints. Differences between techniques were small and only statistically significant between Elastic Net and Logistic Regression compared with Random Forest for 3-years HF hospitalisation.

**Conclusion:**

In this study based on a health insurance claims database we found clear predictive value for predicting long-term HF hospitalisation and mortality of CHF patients by using ML techniques compared to traditional statistics.

**Supplementary Information:**

The online version contains supplementary material available at 10.1186/s12911-021-01657-w.

## Background

Chronic heart failure (CHF) is a severe condition that is characterized by high mortality and morbidity. Evidence exists that a substantial portion of CHF patients, in particular those with (multiple) comorbidities, do not currently receive optimal medical therapy, leading to potentially avoidable specialist-visits and frequent HF hospitalisations, impaired quality of life or even life-threatening complications [1–5]. Patients admitted for CHF are at a considerably higher risk of (long-term) adverse outcomes after a hospital discharge than the general elderly population, even higher than after other common serious events such as pneumonia and myocardial infarction [6]. Accurately predicting which CHF patients are particularly vulnerable for adverse outcomes, such as renewed HF hospitalisation or even death, is of crucial importance to support clinical decision-making. Advances in statistical approaches and computational power, including fully utilizing machine learning techniques on Big Data, potentially provide better knowledge extraction and evidence-based clinical decision support [7–11]. In addition to traditional statistical analysis, novel machine learning (ML) algorithms can identify patterns in large datasets and build both linear and non-linear models in order to make effective data-driven predictions [12]. All residents of the Netherlands are entitled to a comprehensive basic health insurance package and this includes the bulk of essential medical care, medications and medical aids. Health insurance claims (HIC) databases are attractive for research because of their large size, their longitudinal perspective, and their practice-based information. As they are based on financial reimbursement, the information is generally reliable, moreover databases are audited every year to ensure that they meet the required quality level for the Dutch risk equalization model [13, 14]. ML techniques could potentially better utilize the richness of these databases [7, 8, 15, 16]. The goal of the current study was to examine the predictive value of Dutch HIC data on long term HF hospitalisation and all-cause mortality in CHF patients, by exploring and exploiting ML and traditional statistical techniques.

## Methods

### Patients

**Fig. 1:**
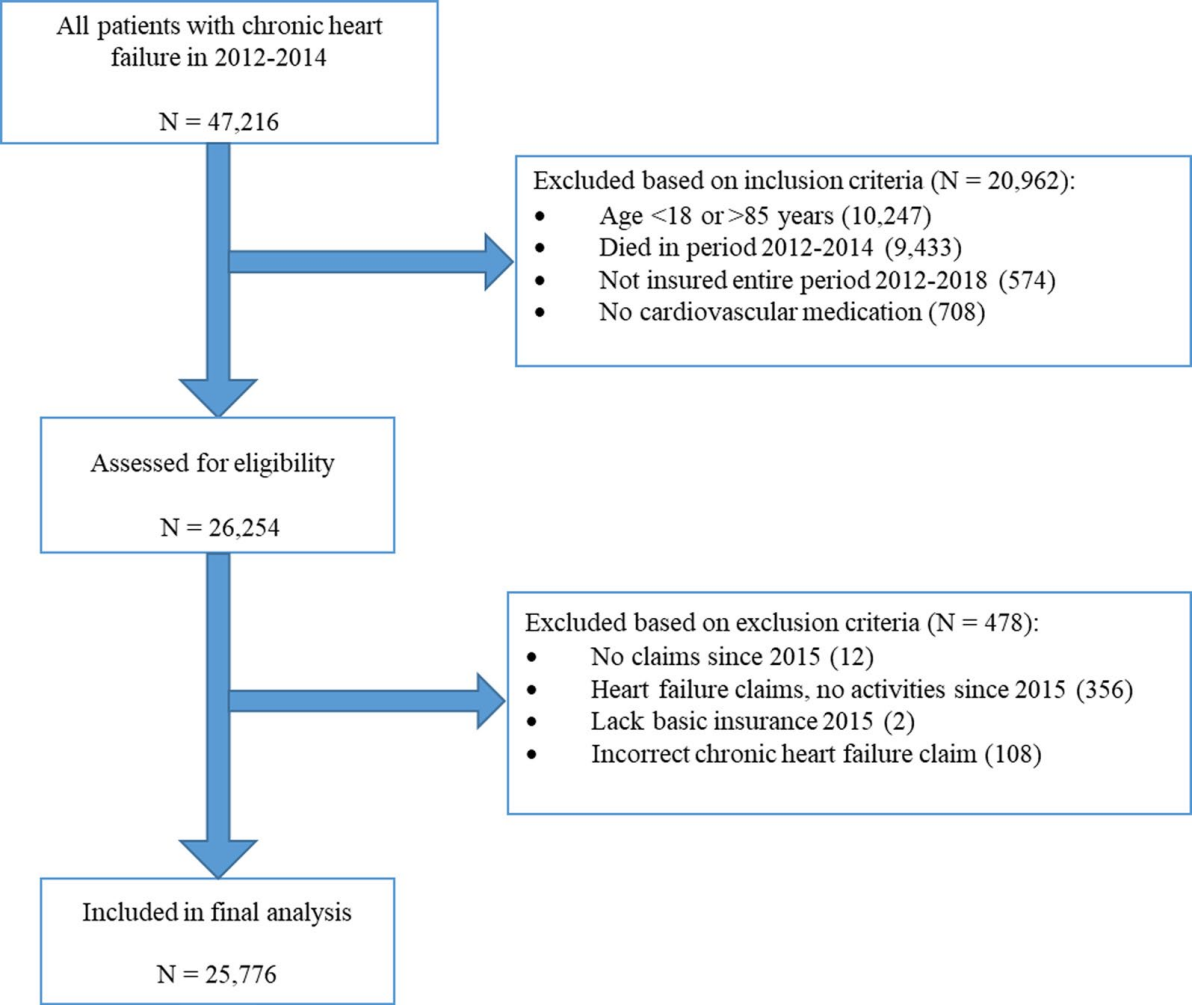
Flowchart of study population. See also: Gürgöze MT, van der Galiën OP, Limpens MAM, Roest S, Hoekstra RC, IJpma AS, Brugts JJ, Manintveld OC, Boersma E. Impact of sex differences in co-morbidities and medication adherence on outcome in 25 776 heart failure patients. ESC Heart Fail. 2020

A HIC database containing anonymous data that can be considered a representative sample of ~ 30% of the Dutch population from Zilveren Kruis, the largest insurance company in the Netherlands, was analysed retrospectively. Patients aged 18–85 years with a diagnosis code for CHF between 2012–2014 were included and follow-up HF hospitalisation and all-cause mortality in 2015–2018 was measured [17]. Patients had to have a CHF-related claim according to the national diagnosis-treatment classification system called ‘Diagnose Behandeling Combinatie’ (DBC), which is based on a combination of the International Classification of Diseases, 10th revision (ICD-10) and applied treatment [18]. Additionally, they had to have used at least one medication within the cardiovascular system (“C”) based on the World Health Organization Anatomical Therapeutic Chemical Classification index and Defined Daily Dose (WHO ATC/DDD) in the same period [19]. According to the European Society of Cardiology (ESC) heart failure guidelines [20], CHF patients should visit their treating physician at least once per year. Therefore, patients were excluded who lacked any HF insurance claim after January 2015, because they are most likely wrongly diagnosed or labelled HF patients. Patients who switched insurance company between 2012–2017 were also excluded. A total of 25,776 patients were included in the final analysis (Fig. 1).

### Endpoints

The study endpoints were HF hospitalisation and all-cause mortality. The risk of HF hospitalisation and all-cause mortality were predicted on a one (2015)- and three-years (2015–2017) perspective. This resulted in the following study endpoints (i.e. dependent features): (1) 1-year HF hospitalisation (1446 patients), (2) 3-years HF hospitalisation (3220 patients), (3) 1-year all-cause mortality (2434 patients), (4) 3-years all-cause mortality (7882 patients). HF hospitalisation was defined as at least one night of stay in inpatient care for acute or chronic HF based on the DBC system. All-cause mortality was defined as death due to any cause. No clinical adjudication committee reviewed the HF hospitalisation endpoint.

### Data

**Fig. 2:**
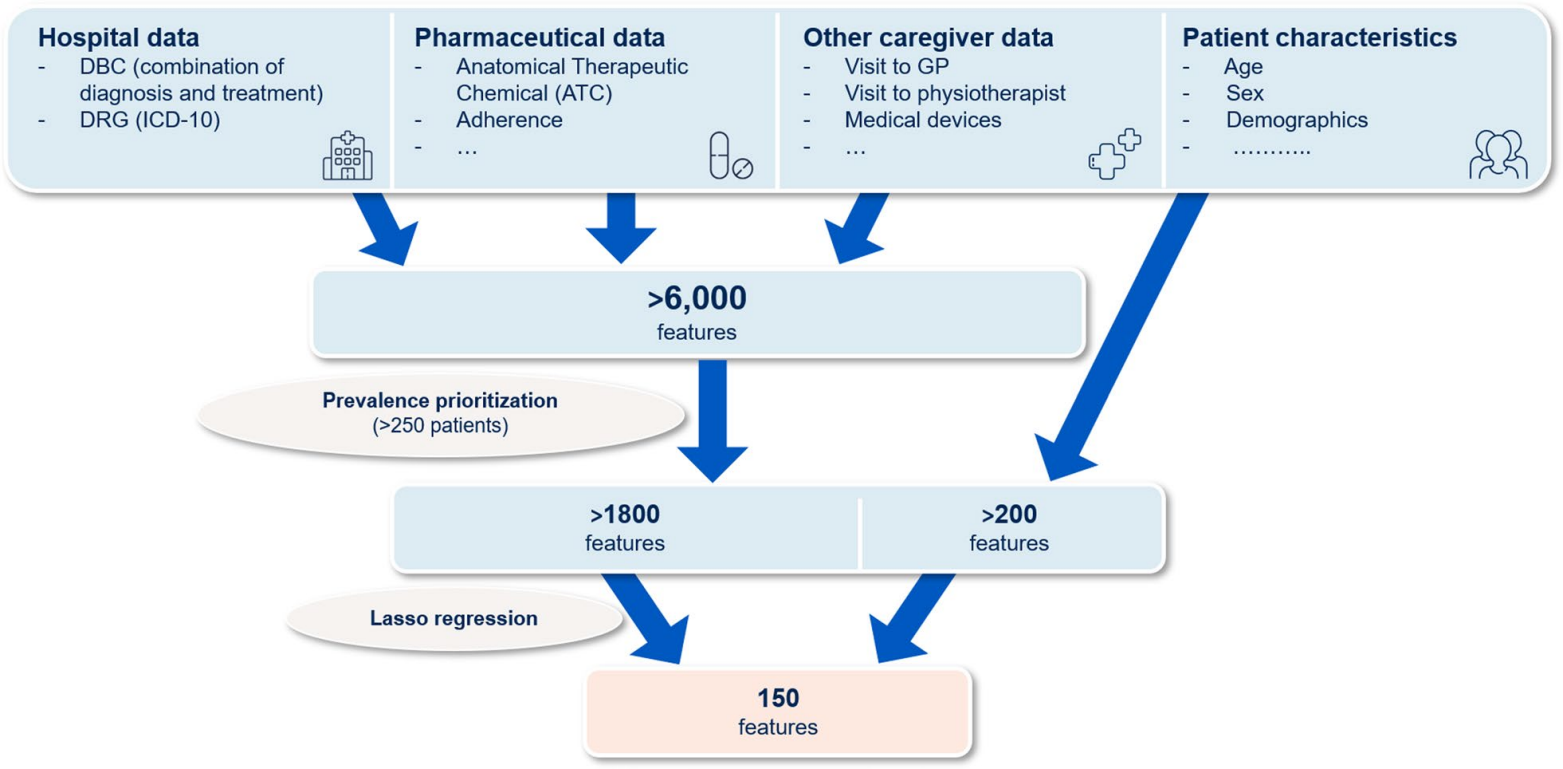
Flow diagram of the process of feature selection

The process of feature selection is graphically displayed in Fig. 2. Claim-based input features between 2012–2014 were divided into three categories: hospital claims, pharmaceutical claims and claims of other caregivers. Hospital claims are all DBC’s, a combination of diagnosis and treatment, for instance a DBC for hospital admission for CHF with more than five nursing days. Within the hospital claims we also included the diagnosis related groups (DRG) based on the ICD-10 code of each DBC. The pharmaceutical claims were divided in seven categories:

1. Use of an individual prescription on a full anatomical therapeutic chemical (ATC) level
2. An ATC3 therapeutic subgroup level [21]
3. Medical adherence by defining the medication possession ratio (MPR) [22, 23]
4. Use of automatic pill dispenser
5. Sum of prescribed daily doses (PDD) which takes into account dosage schemes as prescribed by the treating physician
6. Number of times medicines were collected
7. Number of different medication within the same ATC3 subgroup.

An example of claims of other caregivers are number of visits to a GP or physiotherapist or use of a medical device.

Most features are categorical and are binary coded to represent whether the corresponding medical service was provided to the patient or not. Due to the large number of claim-related features of the dataset (> 6000 features), feature selection plays an important role in reducing noise and computational costs, while simultaneously improving accuracy [24]. Feature selection was done in two stages; first, prevalence prioritization and, second, a Lasso Regression [25]. Prevalence prioritization was performed in each of the three categories and their relevant subcategories for two-time episodes, by first selecting all features, with a threshold of > 250 patients in each category. In this way we included for two-time episodes 96 and 160 out of 2290 hospital claim features, 73 and 85 out of 141 DRG features, 192 and 232 out of 901 pharmaceutical claims features, 55 and 60 out of 299 pharmaceutical claims on a ATC3 level and 377 and 508 out of 3232 other claims features. Input features between 2012–2014 were divided in two-time episodes; (1) year 2014 and (2) combined years 2012–2013. Patient characteristics on postal code level, such as income (high, medium, low) and distance to nearest facilities such as GP and hospital, were also included as input features. Two time-related features were included in the model; days between last hospital visit in 2012–2014 and January 1, 2015, and duration since first hospital visit, by determining the period between the first occurrence of DBC for CHF in this baseline period up to January 1, 2015. The total number of input features in this first stage was > 2000. In the second stage of feature selection we ran a Lasso regression to obtain the (maximum) 150 most significant features on the partition of the dataset that was subsequently used for model training (49%) and validation (21%), on all the input features of stage 1, related to each of the four endpoints separately. The LASSO method puts a constraint on the sum of the absolute values of the model parameters, the sum has to be less than a fixed value (upper bound). In order to do so the method applies L1 regularization, where some of the coefficients become exactly zero. The variables corresponding to the non-zero coefficients remain in the dataset. The goal of this process is to reduce computation cost and was to minimize the prediction error [25]. We used SAS Enterprise Guide 7.1 for Lasso regression (proc GLMSELECT for binary outcome with Schwarz Bayes selection Criterion) [26]. Baseline characteristics age, sex and marital status were added to the final set. The features and the total number of features in the final set is described in Additional file 1: Table I. The definition of demographics, socio-economic status, selection of medication and all other input features are described in Additional file 1: Table II.

### Statistical analysis

We compared four computational techniques to determine which method yields the best prediction for the study endpoints: backward logistic regression (LR), regularized logistic regression (Elastic Net, EN), random forests (RF) and neural networks (NN). We used the area under the receiver operating characteristic (ROC) curve (AUC) as primary and sensitivity and specificity as the secondary performance metric for comparing the models. Sensitivity and specificity have the advantage that they express equal importance to the correct prediction of hospitalisation/mortality and the prediction of no hospitalisation or mortality [27] Additional performance metrics, such as true negatives and precision are calculated and shown in a confusion matrix in Additional file 1: Table III.

The dataset has been split randomly into two partitions, to learn (training and validation) and evaluate (test) the models. The first partition of 70% of the complete dataset (patients) to learn is used for model training (49%) and validation (21%). Various combinations of hyperparameter values (Table 1) were explored to optimize the AUC between training set and validation set to obtain the best trained model. For the hyperparameters not mentioned, the respective default values of the software packages R Statistical Software and SAS Enterprise Guide were used. The second partition of 30% of the complete dataset is then used to evaluate the final prediction performance. The same learning and evaluation partitions were used for all techniques.

**Table 1:** Hyperparameters used in the several models

Backward logistic regression Select criterion: significance level Stay significance level: 0.05	Elastic Net Alpha: 0–1 stepped by 0.1 Lambda*: 0.001 to 100.000 in 80 exponential increasing steps Folds for cross validation: 10
Random forest Max trees Max depth Split criterion: Gini	Neural network Type: fully connected feed forward Architecture: 1–3 hidden layer with 10–100 nodes

^*^The lambda sequence is created using the following formula 10 ^ seq (from =  − 2, to = 5, by = 0.1). This generates 71 values from 0 to 100.000

Because the dichotomous endpoints (hospitalisation and mortality) of the models are imbalanced (> 90% has class value ‘no mortality’, or ‘no hospitalisation), by default the predictions could be biased towards the class that has the highest prevalence [28]. Therefore, the loss is separated per class and the class loss is weighted proportionally to the inverse of the proportion of the corresponding class (formula described in Additional file 1: Table II).

The backward logistic regression (LR) starts with all coefficients in the model and deletes them consecutively. In each step the coefficient that does not (significantly) improve the prediction on the dependent variable is removed until all features have a significance greater than 0.10.

The regularized Logistic Regression was estimated with the elastic net regularization (EN). This is a combination of the LASSO regularization (L1 penalty) and ridge regularization (L2 penalty). Therefore, there are two hyperparameters that need to be tuned: alpha (L1 penalty) and lambda (L2 penalty). The optimal combination of alpha and lambda is searched for with tenfold cross validation on the validation set. For alpha all values between 0 and 1 with an interval of 0.1 are used. For lambda a range between 0.001 and 10.000 is used in 80 exponentially increasing steps. This resulted in 880 combinations of different alpha and lambda values.

The Random Forest model (RF) is an ensemble of multiple decision trees. Each step in the decision tree construction uses a selection of the input features ($\sqrt {total\;number\;of\;variables}$) and per tree a subset of the training data. The splitting criteria is the Gini-index. We let the algorithm infer the optimal number of trees itself such that the misclassification rate on the out-of-bag samples is minimized.

For the neural network (NN) a fully connected feed forward network was used. We explored multiple architectures with a varying number of hidden layers and nodes per layer. Networks with 1–3 hidden layers are optimized with each layer having 10 up to 100 hidden nodes. Only the results of the best architecture optimized on the validation set are presented in the results section.

Cut-off values for all four computational techniques, for the additional performance metrics such as sensitivity and misclassification were derived from the Youden index, which is the sum of sensitivity and specificity minus one [29]. The total number of input features was used for all statistical techniques.

For NN variable importance (VI) is hard to establish because of its “black box” nature [30, 31]. We therefore computed VI only for RF, EN and LR. VI in the RF was calculated based on Random Branch Assignments Variable Importance (RBA). The RBA is evidently much less influenced by correlations [32]. For EN we used the absolute values of the coefficients rank the features in the order of variable importance and for LR we used Multivariate Coefficients Score. For this score we simply calculated the magnitude of the marginal effect of each (non-standardized) predictor, by fitting multivariate LR models and filtering out insignificant coefficients according to the *P*-value “stay threshold”. We report the top 10 features, and their number of patients and univariate odds ratio and discussed the outcome of VI with clinicians.

Output data were analysed using R Statistical Software version 3.4.2 (Vienna, Austria), Caret and GLMNET were used to conduct the EN analysis and SAS Enterprise Guide 7.1 for LR, RF and NN, see Table 2 for software and model information.

**Table 2:** Model information

	Stepwise logistic regression		Random forest		Neural network*		Elastic Net
Software	SAS Enterprise Guide 7.1 Proc HPlogistic		SAS Enterprise Guide 7.1 Proc HPforest		SAS Enterprise Guide 7.1 proc HPNeural		R (caret package)
Select criterion	Significance level	Max trees	100	Type	Fully connected feed forward	Alpha	0–1 in steps of 0.1
Stop criterion	Significance level	Mas depth	30	Number of hidden layers	1	Lambda	0.001 to 100.000 in logarithmic steps
Effect hierarchy enforced	None	Prune threshold	0.1	Number of hidden neurons	10–15	Folds for crossvalidation	10
Entry significance level (SLE)	0.05	Leaf fraction	0.00001	Number of weights	7721	Link function	Binomial
Stay significance level (SLS)	0.05	Category bins	30	Optimization technique	Limited memory BFGS		
Stop horizon	1	Interval bins	100	Maxiter	1000		
		Minimum category size	5	Activation function	Identity		
		Rows of sequence to skip	5				
		Split criterion	Gini				
		Preselection method	Loh				

*Multiple architectures are tested for neural networks. The variants used additional layers (up to three) and more hidden nodes per layer (up to 100). Only the best architecture is presented here

## Results

### Baseline characteristics

Our study population consists of 25,776 CHF patients (median age 74 years (Interquartile Range [IQR] 66–80 years) and 43.7% women) including 1446 patients with HF hospitalisation in 2015 and 3220 in 2015–2017 and all-cause mortality 2434 and 7882, respectively. Baseline characteristics of the overall study sample are described in Table 3.

**Table 3:** Baseline characteristics

Characteristics	All patients *N* = 25,776
Age (years), median (IQR)	74 (66–80)
Sex, *n* (%)	
Men	14,517 (56)
Women	11,259 (44)
Marital status, *n* (%)	
Married	8697 (34)
Unknown	8428 (33)
Widow/widower	3802 (15)
Never married	3040 (12)
Divorced	1809 (7)
SES score, median (IQR)	− 0.37 (− 1.17–0.47)
Income level, median (IQR)	5.0 (2.0–7.0)
Duration since last visit*, *n* (%)	
0–6 months	2993 (12)
6–12 months	3327 (13)
1–2 years	6975 (27)
> 2 years	12,481 (48)
Hospitalisation, *n* (%)	
Year 1 (2015)	1446 (6)
Year 1–3 (2015–2017)	3220 (12)
All-cause mortality, *n* (%)	
Year 1 (2015)	2434 (9)
Year 1–3 (2015–2017)	7882 (31)

*Period between the last occurrence of DBC for CHF in this baseline period up to January 1, 2015

### Performance metrics and relevant features

AUC rates ranged from 0.710 to 0.732 for 1-year HF hospitalisation, 0.705–0.733 for 3-years HF hospitalisation, 0.765–0.787 for 1-year mortality and 0.764–0.791 for 3-years mortality. Elastic Net performed best for all endpoints (Table 4). Differences between techniques were small and only statistically significant between EN and LR compared with RF for 3-years HF hospitalisation, based on the confidence intervals.

**Table 4:** Confusion matrix

	(n = 7733)	AUC*	CI^†^		Sensitivity (%)	Specificity (%)
Logistic Regression	1-Year HF hospitalisation	0.7099	0.6822	0.7375	61.4	73.6
Random forest	1-Year HF hospitalisation	0.7075	0.6815	0.7335	62.3	68.1
Neural network	1-Year HF hospitalisation	0.7319	0.7061	0.7577	71.9	63.9
Elastic net	1-Year HF hospitalisation	0.7320	0.7066	0.7575	71.0	66.3
Logistic regression	3-Years HF hospitalisation	0.7255	0.7088	0.7422	71.5	62.0
Random forest	3-Years HF hospitalisation	0.7045	0.6874	0.7217	73.1	57.8
Neural network	3-Years HF hospitalisation	0.7313	0.7147	0.7479	67.3	68.9
Elastic net	3-Years HF hospitalisation	0.7330	0.7165	0.7495	67.8	67.7
Logistic regression	1-Year all-cause mortality	0.7746	0.7568	0.7923	78.0	63.4
Random forest	1-Year all-cause mortality	0.7649	0.7471	0.7827	59.8	80.1
Neural network	1-Year all-cause mortality	0.7664	0.7483	0.7845	76.7	62.6
Elastic net	1-Year all-cause mortality	0.7866	0.7691	0.8040	74.2	69.7
Logistic regression	3-Years all-cause mortality	0.7897	0.7790	0.8003	79.2	63.1
Random forest	3-Years all-cause mortality	0.7639	0.7527	0.7751	71.8	67.8
Neural network	3-Years all-cause mortality	0.7817	0.7709	0.7925	75.0	67.1
Elastic net	3-Years all-cause mortality	0.7911	0.7805	0.8017	64.4	78.1

*Area under the Curve, ^†^95% confidence interval

Sensitivity ranged from 61.4 to 71.9 for 1-year HF hospitalisation, 67.3–71.5 for 3-years HF hospitalisation, 76.5–78.7 for 1-year mortality and 64.4–79.2 for 3-years mortality. Specificity ranged from 66.3 to 73.6 for 1-year HF hospitalisation, 57.8–68.9 for 3-years HF hospitalisation, 62.6–80.1 for 1-year mortality and 63.1–78.1 for 3-years mortality. For sensitivity rates NN outperformed for 1-year HF hospitalisation and 1-year mortality, while EN outperformed for 3-years HF hospitalisation and LR for 3-years mortality. For specificity LR outperformed for 1-year HF hospitalisation, NN for 3-years HF hospitalisation, RF for 1-year mortality and EN for 3-years mortality.

Top-10 features of importance in our study are shown in Tables 5, 6, 7 and 8. For HF hospitalisation, previous HF hospitalisation for CHF or acute HF, comorbidities as COPD, diabetes or oncology and visit to the GP are the most common among the trained models. For mortality, age, sex and marital status are also often used in the models. Features from 2014 were more common compared to 2012–2013.

**Table 5:** Variable importance 1 year HF hospitalisation

Feature	Description feature	N	Univariate	Random forest	Elastic net	Logistic regression
			OR*	(MSE^†^)	(coefficients)	(predictor)
dbc_2014_099899068	> 5 Nursing days acuut CHF	753	4.5	0.00055	0.66195	0.74370
dbc_2014_099899024	< 6 Nursing days acuut HF	674	3.9	0.00005	0.50642	0.84080
dbc_2014_099899046	< 6 Nursing days CHF	1692	2.7	0.00022	0.48950	
zrg_2014_OX04070489	DBC laboratory research	2656	1.2	0.00015		
zrg_2012_OX02070820	BNP/NT-proBNP Laboratory research	3130	1.2	0.00011		
DRG_2012_C64_C68	Malignant neoplasm of urinary tract	553	1.5	0.00009		
zrg_2014_500112001	Consult GP > 20 min	12,488	1.1	0.00009		
ATC_2014_C09AA02	Enalapril/enalaprilaat	1996	1.3	0.00009		
zrg_2012_U46012300	Consult GP	5086	1.2	0.00006		
dbc_2012_099899003	Surgical team meeting and/or outpatient clinic visit in case of a disease of the heart	1112	1.8	0.00005		
dbc_2012_131999206	Outpatient clinical visit rheumatism	401	0.6		0.57427	1.56740
ATC3_2014_A01	Mouth preparations	280	1.8		0.50668	0.82770
DRG_2012_C81_C96	Malignant neoplasm lymphoid and blood-forming tissue	690	1.5		0.42163	0.72440
ATC_2012_R03AC12	Salmeterol	344	0.8		0.53758	0.70620
zrg_2012_OX04080080	DBC Radiology	269	0.1		0,67,103	
ATC_2014_C09AA01	Captopril	291	0.9		0,46,435	
dbc_2014_099899050	> 5 Nursing days acuut HF	977	4.0		0,43,989	
zrg_2012_701013016	Post-operative consultation inc. removal of suture material, in practice GP aid	602	0.7			1.11550
DRG_2012_C64_C68	Malignant neoplasm of urinary tract	553	1.5			0.99730
dbc_2012_079999013	Outpatient clinic visit eye disease	333	0.8			0.76760
dbc_2012_100501045	Day treatment asthma	353	1.1			0.68920

^*^Odds ratio, ^†^mean square error

**Table 6:** Variable importance 3 years HF hospitalisation

Feature	Description feature	N	Univariate	Random forest	Elastic net	Logistic regression
			OR*	(MSE^†^)	(coefficients)	(predictor)
dbc_2014_099899068	> 5 Nursing days acuut CHF	753	3.4	0.00155	0.59463	0.77700
DRG_2014_I00_I02	Acuut HF	1564	3.0	0.00095	0.56953	0.62340
dbc_2014_099899046	< 6 Nursing days CHF	1692	2.0	0.00059		
ATC_2012_B01AC04	Buildings built between 1965 and 1974	2740	1.6	0.00054		
RRafl_Amiodaron	Number collected medication Amiodaron	1586	1.0	0.00029		
DRG_2014_E10_E13	Diabetes	2517	2.0	0.00024		
zrg_2014_B291079992	Home visit	3539	1.0	0.00019		
zrg_2012_U46012300	Consult GP	5086	1.2	0.00016		
zrg_2012_500112002	Visit GP	8540	1.0	0.00015		
ATC_2012_C03DA01	Spironolacton	7301	2.0	0.00014		
dbc_2012_029899013	Outpatient clinic visit benign growth of the skin	260	0.5		0.53165	− 0.71500
zrg_2014_5603P30	Complete dentures upper and lower jaw Complete dentures upper and lower jaw	504	1.6		0.41897	0.61270
ATC_2014_V03AE02	Sevelameer	304	0.8		0.74320	− 0.58770
DRG_2014_D22_D23	Benign neoplasm skin	362	1.3		0.47009	0.52520
VERHUISD_2015	Movend out in 2015	255	1.3		0.53365	
zrg_2012_OX04084602	DBC Radiology	679	0.8		0.45328	
ATC3_2014_B05	Blood replacement agents	288	0.9		0.44734	
ATC_2014_J02AC01	Fluconazol	251	0.8		0.43146	
ATC_2014_R03BA08	Ciclesonide	570	0.8			− 0.60770
dbc_2014_131999210	Outpatient clinic visit gout	260	2.4			0.56250
dbc_2014_099899091	Outpatient clinic visit heart valve abnormality	399	1.5			0.48490
DRG_2012_T80_T88	Osteoarthritis revision prosthesis	304	1.5			0.47710

*Odds ratio, ^†^mean square error

**Table 7:** Variable importance 1 year all-cause mortality

Feature	Description feature	N	Univariate	Random forest	Elastic net	Logistic regression
			OR*	(MSE^†^)	(coefficients)	(predictor)
oms_burg_staat2	Marital status	25,776	0.7	0.00236	0.84190	
geslacht	Sexe	25,776	1.3	0.00075		
ATC_2014_B03XA02	Darbepoetine alfa	356	4.9	0.00040		
age	Age	25,776	1.1	0.00036		
DRG_2014_C30_C39	Malignant neoplasm intrathoracic	552	3.5	0.00035		
zrg_2014_500112003	Visit GP > 20 min	5932	1.2	0.00031		
zrg_2014_I001196001	Ambulance	5909	1.4	0.00031		
dbc_2014_099899068	> 5 nursing days acuut CHF	753	4.1	0.00028		
zrg_2014_701013035	Visit GP > 20 min	274	1.4	0.00027		
ATC_2014_H02AB06	Prednisolon	4712	2.3	0.00027		
dbc_2014_090301002	Outpatient clinical visit high bloodpressure	270	0.1		0.85177	−1.39220
dbc_2014_099899068	> 5 nursing days acuut CHF	753	4.1		0.74860	1.20540
DRG_2014_C30_C39	Malignant neoplasm intrathoracic	552	3.5		0.92866	0.86620
DRG_2014_R52_1	Pain	359	0.9		0.78378	−0.84680
DRG_2014_C00_C98	Malignant neoplasms—other	584	2.6		0.66214	0.81190
DRG_2014_AULG_01	Audiology	250	0.5		0.75475	
dbc_2012_090301002	Outpatient clinical visit high bloodpressure	567	0.5		0.68019	
zrg_2014_6409120361	Toilet riser device	358	2.4		0.59082	
zrg_2014_6404330361	Seat cushion foam/static seat cushion	315	5.2		0.58836	
dbc_2012_039999015	Outpatient clinic visit blood disease	313	1.8			1.04530
dbc_2012_100501046	Outpatient clinic visit asthma	471	0.7			−0.95950
dbc_2012_099599003	< 6 Nursing days acuut heart disease	277	1.7			0.82520
zrg_2014_OX02089402	Examination with X-ray of the knee and / or lower leg. Radiological examination	577	0.6			−0.77200
DRG_2012_C81_C96	Malignant neoplasm lymphoid and blood-forming tissue	690	1.9			0.74920

*Odds ratio, ^†^mean square error

**Table 8:** Variable importance 3 years all-cause mortality

Feature	Description feature	N	Univariate	Random forest	Elastic net	Logistic regression
			OR*	(MSE^†^)	(coefficients)	(predictor)
dbc_2014_099899068	> 5 nursing days acuut CHF	753	3.4	0,00,036	0.62778	0.82270
Age	Age	25,776	1.1	0,00,501		
Geslacht	Sexe	25,776	1.2	0,00,222		
zrg_2014_500112003	Visit GP > 20 min	5932	1.4	0,00,151		
RRPDD_Loop	PDD Loop	14,166	1.0	0,00,065		
DRG_2012_KGER	Clinical geriatrics	1225	3.2	0,00,064		
ATC_2014_A03FA01	Metoclopramide	1059	2.4	0,00,062		
DRG_2014_C30_C39	Malignant neoplasm intrathoracic	552	3.0	0,00,060		
oms_burg_staat2	Marital status	25,776	0.7	0,00,044		
DRG_2014_C00_C98	Malignant neoplasms—other	584	2.1	0,00,035		
dbc_2014_100501025	> 5 nursings days COPD	377	5.5		0.60695	0.92830
DRG_2014_C30_C39	Malignant neoplasm intrathoracic	552	3.0		0.71789	0.88990
DRG_2014_C00_C98	Malignant neoplasms—other	584	2.1		0.64685	0.71970
DRG_2014_M16	Osteoarthritis hip	320	0.6		0.58461	− 0.62770
ATC3_2014_V03	Other therepeutic devices	475	3.6		0.49412	0.61010
ATC_2014_R03DC03	Montelukast	253	0.8		0.72986	− 0.90920
ATC_2012_N05AD01	HALOPERIDOL	363	3.8		0.71630	0.72350
DRG_2014_J80_J84	Interstitial lung disease	298	1.9		0.73885	0.72180
ATC3_2014_L01	Oncolytica	415	1.9		0.52782	
ATC_2014_B03XA02	Darbepoetine alfa	356	4.9			0.62320

*Odds ratio, ^†^mean square error

## Discussion

In this analysis, based on a HIC database of > 25,000 patients with CHF, we have shown that the use of traditional and novel techniques indeed have clear predictive value for predicting long-term HF hospitalisation and all-cause mortality for CHF patients, with AUC’s between 0.7 and 0.8 [33, 34].

For our main performance metric, the AUC, EN outperformed other statistical methods in predicting 1 and 3 years HF hospitalisation and 1 and 3 years mortality, although with only minor differences compared to traditional LR and only statistically significant between EN and LR compared with RF for 3-years HF hospitalisation.

Our results are comparable with earlier reported findings. Angraal et al. reported in a recent study for 3-years mortality and HF hospitalisation that RF was the best performing model with a mean AUC of 0.72 (95% confidence interval [CI] 0.69–0.75) for predicting 3-years mortality, and 0.76 (95% CI 0.71–0.81) for 3-years HF hospitalisation [35]. This study was based on a cohort with 1,767 patients in HF with preserved ejection fraction. Chicco et al. [36] analysed 9 months mortality in 299 patients with HF. RF outperformed all the other methods, by obtaining the top ROC AUC (0.800) The Artificial Neural Network perceptron, instead, obtained the top value on the Precision-Recall AUC (0.750). The AUC outcomes on our HIC database are in line with these 2 recent studies, both based on clinical data, although RF did not outperform in our study. Mortzavi et al. found more pronounced differences (10–25%) for 30-days HF hospitalisation and 180-days HF hospitalisation outcome between ML compared to traditional LR, but they only used the 5 most important features as identified previously (blood urea nitrogen, glomerular filtration rate, sex, waist-to-hip ratio, and history of ischemic cardiomyopathy) in LR and the remaining techniques were created using the full raw data of 472 inputs [37]. It should be noted that these studies had small datasets, which may limit the generalizability of their conclusions. A meta-analysis and meta-regression study of 117 prognostic models revealed only a moderate accuracy of models predicting mortality, whereas models designed to predict the combined endpoint of death or HF hospitalisation, or only HF hospitalisation, had an even poorer discriminative ability. The highest AUC-statistic values were achieved in a clinical setting, predicting short-term mortality with the use of models derived from prospective cohort/registry studies with many predictor variables. The mean AUC-statistic was 0.66 ± 0.0005 for the models reporting a standard error. Models using data from medical records had significantly better AUC-statistic values than models using claims data. Also, models using more predictor variables had better predictive values; AUC-statistic increased 0.0036 (SE = 0.0005) with each added predictor variable. There was no significant difference in AUC-statistic values between patients diagnosed with either CHF or acute HF [38].

Most currently existing prognostic models in patients with CHF are based on data from randomized controlled trials or extracted from administrative datasets, such as medical insurance claims [39]. To our knowledge, this was the first study that applied machine learning techniques to a (Dutch) HIC database for CHF outcome prediction. A great advantage of a HIC database in the Netherlands is that it covers the entire healthcare utilization since over 99% of the population has basic health insurance as mandated by law [40]. Most studies are based on data of 1 or a limited number of hospitals, but in a HIC database we have data of all the hospitals and patient visits. Moreover, a HIC database also includes General Practitioners (GP), pharmaceutical and other healthcare-related data. The relevance of covering a patient’s full healthcare usage was demonstrated by our feature importance analysis (Tables 5, 6, 7, 8) that shows that GP and pharmaceutical data are related to the endpoints in our study. Primary care plays a central role in many countries, such as the Netherlands and the UK in the diagnosis, long-term management, and end-of-life care for these patients. While there is specialist support available from nurses and cardiologists especially after admission and later on at a regular basis once or twice a year, GPs remains responsible for overseeing most patient care once a diagnosis is made including management to delay progression, recognition of HF decompensations, and patient follow-up in the vulnerable period following an HF hospitalisation [41, 42].

Most machine learning techniques for adverse outcomes in CHF focus on a short time period, mainly on 30-days HF hospitalisation [6]. Although the risk for HF hospitalisation declines over time, patients with a CHF hospitalisation have a significantly elevated risk of HF hospitalisation for at least 1 year [4]. From a public health and patient perspective long-term adverse outcomes are as important and a HIC database has adequate information to examine this long-term perspective.

The most important features in our study are well known and reported in earlier studies, for instance age, sex, comorbidities as diabetes or COPD, and living in buildings built between the years 1965 and 1974 as a proxy for socio-economic status. HF Hospitalisation in the baseline period was unsurprisingly an import predictor of HF hospitalisation in the future, as well as Acute HF. Visit of the GP was an import predictor, but we do not know the reason of this visit. Most likely it was related to comorbidities. Importantly, most data-driven machine learning techniques, including the ones we used, are correlational in nature and not causal, so caution while interpreting these results is advised [43].

As the current study was only utilizing HIC data, potentially important clinical features are not included in the current models. For instance, Parenica et al. [44] found higher age, LV dysfunction, comorbidities and high levels of natriuretic peptides as the most powerful predictors of worse prognosis in long-term survival. Reduced ejection fraction is a powerful predictor of long-term mortality, especially after the 6th year [45]. Ouwerkerk et al. [38] found 3 variables with a high predictive value: sodium; blood urea nitrogen; and systolic blood pressure. Clinical features such as ejection fraction and natriuretic peptides are not available in a HIC database, but age and comorbidities based on pharmacy-based cost groups or DRGs are.

We have given an overview of model performance of several machine learning algorithms and traditional statistics in predicting risk for HF hospitalisation and all-cause mortality in a representative sample of the Dutch population from a HIC database. Our findings are therefore generalisable to all CHF patients in the Netherlands. However, several limitations should also be acknowledged. First, models based on administrative claims data lack certain clinically relevant features, such as New York Health Association Class [46], left ventricular ejection faction, intoxications such as smoking, blood pressure and physical activity [47]. Enriching HIC data through clinical data coupling would be preferred. Strict general data protection regulation (GDPR) rules limit these possibilities. Using a trusted third party or novel techniques like Multi Party Computation could provide a good solution to overcome the legal burdens to clinical and HIC data coupling [16]. Second, due to GDPR regulations, patient data history is limited to 7 years in HIC databases, hence, relevant historical data may be missing. Third, the ability to explain and interpret ML models is limited, especially NN. Hence, it is difficult to embrace these models and apply them in a clinically relevant way. More research is needed to explore the causal relationship of features that could be of importance in medical practice. In general, we found poor or moderate overlap between methods in their assessment of feature importance for the top10 features, even when their performance is comparable and relatively good. Most overlap was between EN and LR and least with RF, because RF was heavily nonlinear. The ability to explain and interpret RF is most elaborate, because RF has an integrated procedure of producing variable importance’s [48]. However, for LR we used the multivariate method. This is the simplest feature importance measure tested, and unsurprisingly has strong assumptions, namely that a predictor’s importance is independent of all other factors. It is also important to note that significant predictors in LR may not make useful predictions [49]. Finally, we reduced the input claims features using prevalence prioritization and Lasso Regression, due to performance reasons. By doing so we perchance excluded features which could have been relevant.

In this study based on a Health Insurance Claims database we have shown clear predictive value for predicting long-term HF hospitalisation and mortality of CHF patients. Novel machine learning techniques like RF and NN can obviate more redundant HF hospitalisation or mortality, because they allow for non-linear relations or in the case of EN can reduce irrelevant features. In the long run, we hope that applying state-of-the-art machine learning on clinical data combined with HIC data can improve risk stratification and prognosis by offering high-risk patients timely intervention through for example cardiac rehabilitation and by optimizing medical therapy and stimulating medical adherence.

## Supplementary Information

**Additional file 1:** Overview of the number of features in the final set, definitions used and additional performance metrics.

## Data Availability

The data generated and analysed during the current study are not publicly available due to the privacy of individuals that participated in the study, but are available from the corresponding author on reasonable request.

## Abbreviations

ATC: Anatomical therapeutical chemical classification
AUC: The area under the receiver operating characteristic curve
CHF: Chronic heart failure
DBC: Diagnose behandeling combinatie or diagnosis-treatment combination
DDD: Defined daily dose
DRG: Diagnosis related groups
EN: Elastic net regression
ESC: European society of cardiology
GDPR: General data protection regulation
GP: General practitioner
HF: Heart failure
HIC: Health insurance claims databases
ICD-10: International classification of diseases
IQR: Interquartile range
LR: Logistic regression
ML: Machine learning
MPR: Medication possession ratio
NN: Neural networks
PDD: Prescribed daily doses
RBA: Random branch assignments variable importance
RF: Random forest
ROC: The area under the receiver operating characteristic
VI: Variable importance
