# Supplementary material for: Prediction of long-term hospitalisation and all-cause mortality in patients with chronic heart failure on Dutch claims data: a machine learning approach
Source: BMC Med Inform Decis Mak. 2021 Nov 1;21:303. doi: 10.1186/s12911-021-01657-w (PMC8561992; doi:10.1186/s12911-021-01657-w)
Supplement: Supplementary file 1 — Additional file 1: Overview of the number of features in the final set, definitions used and additional performance metrics. [file 12911_2021_1657_MOESM1_ESM.docx]

Supplementary Material

This appendix has been provided by the authors to give readers additional information about their work.

Supplement to:

**Prediction of long-term hospitalisation and all-cause mortality in patients with Chronic Heart Failure on Dutch claims data: a machine learning approach**

Onno P. van der Galiën^1^, René C. Hoekstra^1,2^, Muhammed T. Gürgöze^3^, Olivier C. Manintveld^3^, Mark R. van den Bunt^1^, Cor J. Veenman^4,5^, Eric Boersma^3^

^1^Zilveren Kruis Achmea, Leusden, The Netherlands

^2^Equalis Strategy & Modeling, The Netherlands

^3^Departement of Cardiology, Erasmus University Medical Centre, Rotterdam, The Netherlands

^4^TNO, The Hague, The Netherlands

^5^Leiden Institute of Advanced Computer Science, Leiden University, Leiden, The Netherlands

**Supplementary material**

**Supplementary Table I. Number of features in the final set**

|  |  | 1-year hospitalisation | 3-years hospitalisation | 1-year mortality | 3-years mortality |
| --- | --- | --- | --- | --- | --- |
| pharmacy full ATC | 2012-2013 | 11 | 17 | 11 | 12 |
|  | 2014 | 22 | 22 | 14 | 15 |
| pharmacy ATC3 | 2012-2013 | 3 | 6 | 4 | 6 |
|  | 2014 | 2 | 5 | 8 | 6 |
| DBC | 2012-2013 | 11 | 13 | 14 | 6 |
|  | 2014 | 14 | 12 | 13 | 9 |
| DRG | 2012-2013 | 7 | 7 | 10 | 12 |
|  | 2014 | 7 | 11 | 11 | 19 |
| other caregivers | 2012-2013 | 33 | 19 | 18 | 21 |
|  | 2014 | 28 | 22 | 36 | 28 |
| Demographics |  | 1 | 3 | 3 | 4 |
| number of times medicines were collected |  | 2 | 1 | 2 | 3 |
| number of different medication within the same ATC3 subgroup |  | 4 | 3 | 1 | 2 |
| PDD |  | 2 | 2 | 1 | 2 |
| Medication Possession Ratio |  | 2 | 3 | 3 | 3 |
| use automatic pill despenser |  |  | 1 |  | 1 |
| Age |  | 1 | 1 | 1 | 1 |
| Sexe |  | 1 | 1 | 1 | 1 |
| marital status |  | 1 | 1 | 1 | 1 |
|  |  | 152 | 150 | 152 | 152 |

**Supplementary Table II. Definitions**

| **Selection of CHF patients** | In the Netherlands, providers are paid through a diagnostic‐related groups system. Such groups are called “Diagnosis Treatment Combinations” (DBCs) (Kroneman M, Boerma W, van den Berg M, Groenwegen P, de Jong J, van Ginneken E. Netherlands *Health system review. European Observatory on Health Systems and Policies 2016; 2016.)*In the period of January 1^st^ 2012 until December 31^st^ 2014 patients with chronic heart failure (CHF) were identified using the following Diagnose Behandeling Combinatie (DBC) codes:  099899045  099899046  099899066  099899067  099899068  Additionally, patients had to have used at least one medication within the cardiovascular system, classified as C based on World Health Organization Anatomical Therapeutic Chemical Classification index and Defined Daily Dose (WHO ATC/DDD) in the same period.  If one of the above CHF DBC codes was registered at least once AND at least one of the medication in class C of WHO ATC/DDD in the given time period, the patient was considered a CHF patient. |
| --- | --- |
| **Selection of hospital admissions** | Acute or chronic heart failure related hospital admissions during the follow-up period from January 2015 until April 29, 2018 were determined using the following DBC codes:  099899049  099899024  099899050  099899108  099899045  099899067  099899046  099899068  099899066  Detailed information of health activities linked with the DBC codes were available from 2015 onwards, including nursing days. These were used to determine hospitalisation. |
| **In Selection of mortality** | Health insurers periodically receive reports of death at an individual level from the Municipal Population Registry (GBA). |
| **Definition demographics & socio-economic status** | Statistics Netherlands (CBS) offers key figures at a low regional level. It concerns data to the full zip code (PC6), 5 digits of the zip code (PC5) and the numerical part of the zip code (PC4). At the most detailed level, these are approximately 450,000 individual zip codes. A range of demographic and socio-economic variables is linked to each area. We used the average distances for residents of the Netherlands from their home address to the nearest medical facilities ( general practitioner, hospital and pharmacy) and information (%) about Social Economic Status, income, residence and ethnicity. |
| **Selection of ATC medication** | Selection of heart failure medication and other medication was done using the World Health Organization Anatomical Therapeutic Chemical Classification index and Defined Daily Dose (WHO ATC/DDD). Usage of combination medications was assigned to each group included in the combination. |
| **Definition of medication adherence** | Medication use and adherence was determined for the period 2012-2014. Medication possession ratio (MPR)9 was defined as the amount of pills corrected for different dosage schemes, the prescribed daily dose (PDD) supplied, divided by the time (days) between two supply dates. Patients may switch drugs within the same class. Therefore, to get more reliable MPR estimates, medications were grouped into ATC classes. We averaged consecutive MPR over the total supply period per ATC group and used categorisation based on a threshold of 0.80, above which a patient was considered adherent to prescribed medication.  The obtained refill rates were categorized in four groups:   1. Adherent; used medication in ATC group and MPR ≥ 0.80 2. Non-adherent; used medication in ATC group and MPR <0.80 3. Unknown; patient used medication, but:    1. Medication was supplied two or less times    2. Prescribed daily dose was not reliable   Never used; patient has never used this medication in 2012-2014 |
| **Selection of hospital claims/DRG** | In the Netherlands, hospitals are reimbursed based on fixed prices for a combination of diagnosis and treatment: a DBC (Dutch: Diagnose Behandeling Combinatie). This hospital funding model has been introduced in January 2005. One of the most important drivers of the introduction of the system was to increase transparency on price and performance of the Dutch (hospital) healthcare. For more information see (in Dutch) <https://www.zorgwijzer.nl/faq/dbc>. Based on the DBC codes Achmea made an internal classification in so called Diagnose Related Groups based on the ICD10 classification. |
| **Selection of other claims (General Practitioner, Physiotherapy, Medical Devices, Oral Health and Medical Transport.)** | The benefit package of the basic health insurance under the Health Insurance Act 2015 consisted of: • medical care, including care provided by GPs, hospitals, medical specialists and midwives. GPs are paid by a combination of fee-for-service, capitation, bundled payments for integrated care, and pay-for-performance (focused on issues such as accessibility and referral patterns).  • home nursing care and personal care (assistance with eating, dressing, etc.).  • dental care for children until the age of 18. For older people only, specialist dental care and a set of false teeth are covered.  • medical aids and devices.  • pharmaceutical care.  • maternity care (midwifery care and maternity care assistance).  • transportation of sick people by ambulance or taxi.  • professions additional to medicine (allied healthcare): physiotherapy for persons with a chronic medical condition (the first 20 sessions relating to the condition are excluded. there is a limiting list of conditions) and for children below the age of 18. occupational therapy. exercise therapy and dietary advice to a limited extent. speech therapy. Health systems in transition The Netherlands 73  • quit-smoking programmes.  • geriatric rehabilitation care.  • care for people with sensory disabilities. and  • mental care: ambulatory mental care and inpatient mental care for the first three years. (After three years inpatient mental care is considered long-term care and is financed by the Long-term Care Act (Wlz).) For some treatments, there are exclusions from the basic insurance package:  • for allied healthcare, generally, a maximum number of sessions are reimbursed.  • some elective procedures, for instance cosmetic plastic surgery without a medical indication, are excluded.  • in vitro fertilization: only the first three attempts are included.  (Kroneman M, Boerma W, van den Berg M, Groenwegen P, de Jong J, van Ginneken E. Netherlands *Health system review. European Observatory on Health Systems and Policies 2016.)*  The Supplementary Insurance covers medical cost that are not (fully) covered by the Basic Health Insurance, like the cost of Dental Care, glasses, Alternative Healthcare, or to cover personal contributions for services or medication that are only partly covered. Basic Health Insurance is mandatory for all citizens and supplementary is not, but 70-80% of the citizens have an supplementary health insurance |
| **Calculation of the weight** | The weight is calculated with the following formula: w_j_=n/(k∗n_j_) where w_j_ is the weight for outcome value j, n is the total number of observations, k is the number of outcome classes (2) and n_j_ is the number of observations in class j. |

**Supplementary Table III. Confusion Matrix additional metrics**

|  | **(n=7,733)** | **True negatives** | **False positives** | **False negatives** | **True positives** | **Mis**  **classification** | **False negattives (%)** | **Precision (%)** | **precision@10** |
| --- | --- | --- | --- | --- | --- | --- | --- | --- | --- |
| Logistic Regression | 1-years HF hospitalisation | 5390 | 1934 | 158 | 251 | 27.1% | 2.04% | 11.5% | 5 |
| Random Forest | 1-years HF hospitalisation | 4990 | 2334 | 154 | 255 | 32.2% | 1.99% | 9.85% | 2 |
| Neural Network | 1-years HF hospitalisation | 4678 | 2646 | 115 | 294 | 35.7% | 1.49% | 10.0% | 3 |
| Elastic Net | 1-years HF hospitalisation | 5200 | 2.24 | 138 | 271 | 30.3% | 1.78% | 11,32% | 3 |
|  |  |  |  |  |  |  |  |  |  |
| Logistic Regression | 3-years HF hospitalisation | 4208 | 2574 | 271 | 680 | 36.8% | 3.50% | 20.9% | 4 |
| Random Forest | 3-years HF hospitalisation | 3919 | 2863 | 256 | 695 | 40.3% | 3.31% | 19.5% | 3 |
| Neural Network | 3-years HF hospitalisation | 4675 | 2107 | 311 | 640 | 31.3% | 4.02% | 23.3% | 6 |
| Elastic Net | 3-years HF hospitalisation | 4601 | 2181 | 307 | 644 | 32.2% | 3.97% | 22.8% | 3 |
|  |  |  |  |  |  |  |  |  |  |
| Logistic Regression | 1-years all-cause mortality | 4434 | 2562 | 162 | 575 | 35.2% | 2.09% | 18.3% | 7 |
| Random Forest | 1-years all-cause mortality | 5601 | 1395 | 296 | 441 | 21.9% | 3.83% | 24.0% | 9 |
| Neural Network | 1-years all-cause mortality | 4377 | 2619 | 172 | 565 | 36.1% | 2.22% | 17.7% | 7 |
| Elastic Net | 1-years all-cause mortality | 5192 | 1804 | 223 | 514 | 26.2% | 2.88% | 22.2% | 7 |
|  |  |  |  |  |  |  |  |  |  |
| Logistic Regression | 3-years all-cause mortality | 3396 | 1983 | 490 | 1.864 | 32.0% | 6.34% | 48.5% | 9 |
| Random Forest | 3-years all-cause mortality | 3647 | 1732 | 663 | 1.691 | 31.0% | 8.57% | 49.4% | 10 |
| Neural Network | 3-years all-cause mortality | 3610 | 1769 | 589 | 1.765 | 30.5% | 7.62% | 49.9% | 10 |
| Elastic Net | 3-years all-cause mortality | 3466 | 1913 | 516 | 1838 | 31.4% | 6.67% | 49.0% | 9 |
